# Characterization of Highly Branched Dextran VB113 Produced by *Leuconostoc mesenteroides* VB113 as Traditional Pickle Isolate

**DOI:** 10.3390/molecules31162763

**Published:** 2026-08-08

**Authors:** Hümeyra İspirli, Akif Emre Kavak, Enes Dertli

**Affiliations:** 1 Food Engineering Department, Engineering Faculty, Bayburt University, Bayburt 69000, Turkey; 2 Nuvita Biosearch R&D Center, Istanbul 34522, Turkey; akif.kavak@nuvita.com.tr; 3 Department of Food Engineering, Faculty of Chemical and Metallurgical Engineering, Istanbul Technical University, Istanbul 34467, Turkey

**Keywords:** Lactic Acid Bacteria (LAB), traditional isolates, homopolymeric exopolysaccharides (HoEPS), characterization of EPS, highly branched dextran

## Abstract

Lactic Acid Bacteria (LAB) from traditional fermented foods serve as a promising source of novel exopolysaccharides (EPSs) with desirable techno-functional properties. In this study, the EPS production ability of LAB strains from traditional pickles was tested and a strong slimy isolate grown in a sucrose-rich environment was further selected. The isolate was identified as *Leuconostoc mesenteroides* VB113 and its EPS production characteristics were determined. HPLC analysis revealed that glucose was the only monomer in the EPS repeating structure, whereas ^1^H and ^13^C NMR spectroscopy analysis demonstrated a branched dextran structure with 20% (1 → 3)-linked α-D-glucose units. Dextran VB113 showed a molecular weight of 2.4 × 10^6^ Da tested by GPC analysis. The functional groups within dextran VB113 were determined by FTIR analysis, and XRD analysis demonstrated its amorphous physical status. TGA and DSC analysis were applied for thermal characterization and a degradation temperature of 272.25 °C was observed for dextran VB113, suggesting its high thermal resistance. The micro-structural features of dextran VB113 were tested by SEM and AFM analysis that revealed spherical and fibrillary properties, respectively. These findings revealed the importance of testing different niches to obtain novel EPS-producing LAB strains that can be further evaluated to enhance the techno-functional properties of food systems. The branched nature of dextran VB113, together with its molecular weight and thermal characteristics, offers practical advantages for its potential usage as a thermal-stable thickening agent, emulsion stabilizer, and fat replacer in high-temperature food processing applications, such as bakery products, processed dairy, and plant-based meat analogs.

## 1. Introduction

Traditional fermented foods are good reservoirs for Lactic Acid Bacteria (LAB), which can be further tested for the production of distinct metabolites that play critical roles in the development of food systems with superior characteristics in terms of technological and functional expectations [1]. Exopolysaccharides (EPSs) are among the critical metabolites of LAB that have been shown to act as potential prebiotics [2], as well as agents that enhance physicochemical properties, such as viscosity and the textural features of food products [3], while also playing roles in probiotic functionality [4]. In terms of structural features, two types of EPSs are produced by LAB strains, categorized as homopolymeric and heteropolymeric EPSs, where the former comprises only one type of sugar monomer in its repeating unit while the latter can contain two or more types of monomers [1]. So far, studies have shown that both homopolymeric and heteropolymeric EPSs could be effective for technological and functional purposes [5,6], although the former has gained special interest due to its low-cost and high-yield production status, as cheap sucrose is the main substrate for its production [7]. LAB strains were shown to produce glucan- and fructan-type homopolymeric EPSs using sucrose as the substrate, and the main hompolymeric EPS for LAB strains is the α-glucan structure [8,9]. Glucansucrases (GSs) are the extracellular enzymes that are responsible for the homopolymeric α-glucan production in LAB, and GSs hydrolyze sucrose into glucose and fructose and a polymerization reaction occurs where glucose units can be linked as (1 → 3), (1 → 6), (1 → 2), or (1 → 4)-linked α-d-glucose units [10]. The proportion of α-d-glucose units with different glycosidic linkages is critical for the characteristics of these glucans, determining their structural type, and so far, four types of α-glucans have been established as alternan, mutan, reuteran and dextran, comprising alternating (1 → 6)/(1 → 3)-linked α-d-glucose units, mainly (1 → 3)-linked α-D-glucose units, mainly (1 → 4)-linked α-d-glucose units, and finally, mainly (1 → 6)-linked α-d-glucose units, respectively [1,9]. Recently, potential subgroups of glucansucrases such as branching sucrases were determined and encoded in certain LAB from distinct niches such as bee-related environments [11], suggesting the importance of identification of LAB strains from different environments to unveil potential new homopolymeric α-glucan producers. Previously, this issue was discussed for sourdough environments, and sourdough-associated LAB strains were assigned to homopolymeric α-glucan production [8]. Similar to the sourdough environment, fermented pickles, as one of the main plant-based fermented foods consumed worldwide with an increasing consumption trend, can be an important reservoir for the LAB strains capable of homopolymeric EPS production, potentially due to the presence of sucrose in the environment [1]. The EPS production capacity of these LAB strains conducting the fermentation process to enhance microbiological, nutritious and physicochemical characteristics might also be important for their starter culture capacity [12], and more studies are required to unveil the α-glucan production capability of pickle-based LAB strains. The unique biological environment of pickles, a primary fermented food product produced through spontaneous household fermentation and stored seasonally under low-pH conditions, could be a key factor driving the presence of high-yield extracellular polysaccharide-producing strains. From these perspectives, the aim of this study was to isolate an EPS producer pickle-based LAB strain and to characterize this EPS in terms of structural and physicochemical features. For this, *Leuconostoc mesenteroides* VB113 was selected among the isolates due to its slimy characteristics in sucrose-rich agar environments. EPS VB113 was further extracted, partially purified and subjected to HPLC analysis to determine its repeating unit structure. For the structural characterization, ^1^H and ^13^C NMR spectroscopy analyses were applied, followed by FTIR analysis to detect the functional groups within the EPS structure. GPC and XRD analyses were applied to determine the final molecular weight and physical status of EPS VB113, whereas TGA and DSC analysis were performed to unveil its thermal properties. Finally, the morphological features, including surface topography, were determined by SEM and AFM analyses. The results of this study may enhance our understanding of the production of different EPSs by distinct LAB strains originating from fermented plant-based environments, potentially leading to the identification of both the EPS and the producer strain for future applications.

## 2. Results and Discussion

### 2.1. Leuconostoc Mesenteroides VB113 as an EPS Producer Pickled Isolate

**Figure 1:**
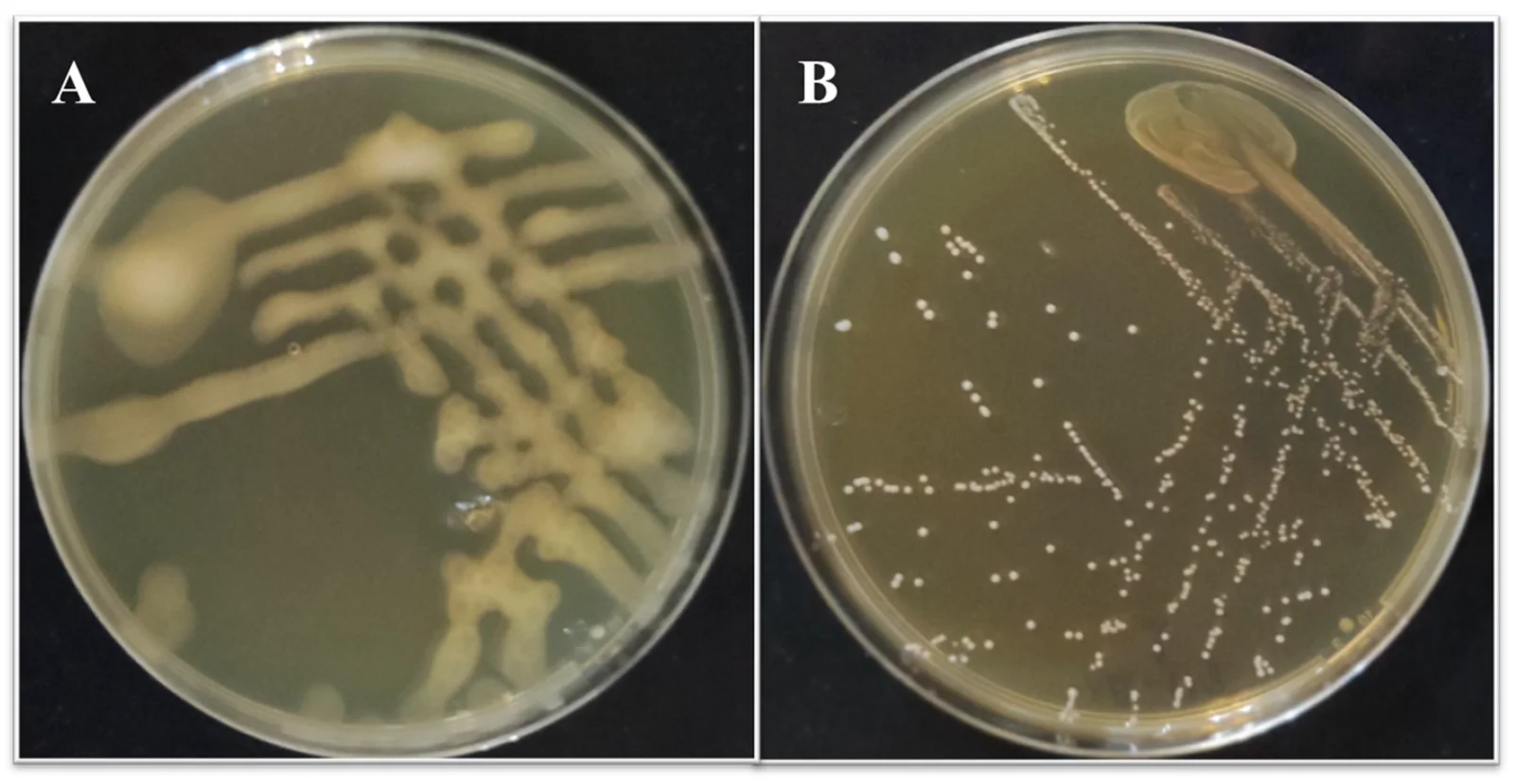
*Leuconostoc mesenteroides* VB113 was capable of slime colony formation in the presence of sucrose (**A**) but not in its absence (**B**).

Sucrose-rich environments such as pickled vegetables or fruits can be rich environments for the isolation of homopolymeric EPS-producing LAB strains [1], and *Leuconostoc mesenteroides* VB113 was previously isolated from traditional Caucasian Pear pickles as a potential EPS producer. The pH of the pickle samples was in the range of pH 3.5 and the samples were spontaneously fermented for 4 months before consumption. Presence of sucrose in the growth medium can be a determinant factor for the EPS production capability of isolates and, as can be seen in Figure 1A, EPS producer strains can form a strong slimy profile, whereas no slimy profile can be observed when sucrose is absent in the agar medium (Figure 1B). Similar to our observation, previous studies demonstrated the isolation of EPS producer strains from pickle environments, suggesting the suitability of the pickle environment for isolation of EPS-producing LAB strains [1,13,14,15]. Fruit-based pickles can be produced traditionally, and this data suggests the potential of this environment as a reservoir for LAB with EPS production capabilities. Following the characterization of the slimy profile in the sucrose-rich environment, *Leuconostoc mesenteroides* VB113 was grown in modified BHI broth containing sucrose and subjected to EPS extraction process. The EPS yield of *Leuconostoc mesenteroides* VB113 was found to be 5.01 g L^−1^ under the tested conditions, but this level can be optimized depending on culture conditions, as in previous studies, a higher level up to 50 g L^−1^ of EPS production was reported for other *Leuconostoc* spp. [16].

### 2.2. Determination of the Repeating Unit and Molecular Weight of EPS VB113

**Figure 2:**
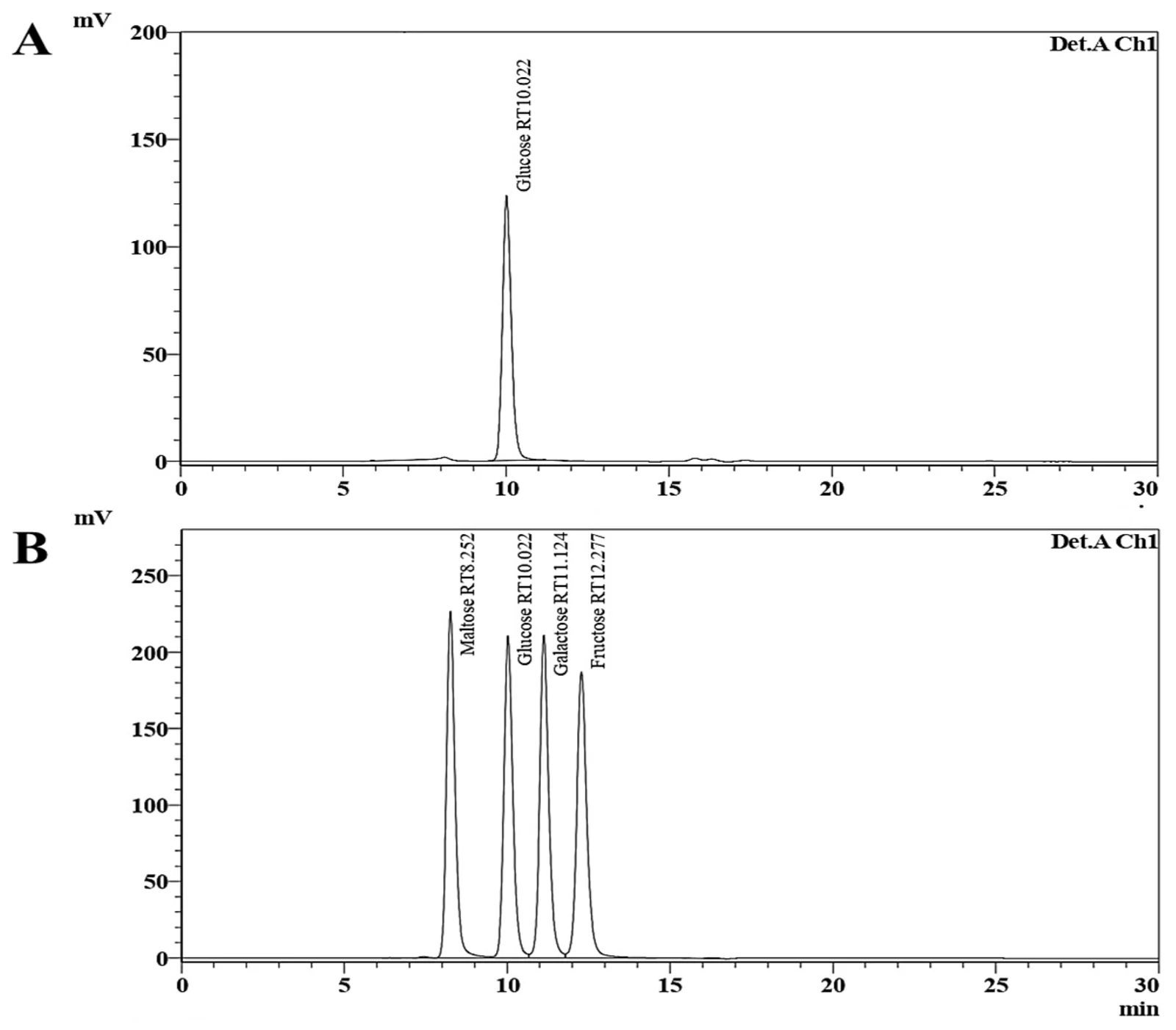
HPLC chromatogram of EPS VB113 (**A**) and the standard sugars (**B**) showing the formation of only glucose in the EPS repeating unit of VB113.

The main structural type for the EPS production characteristics of *Leuconostoc mesenteroides* strains is the production of homopolymeric α-glucans [9]. Having a strong slimy profile for strain VB113, at first, we determined the monomer unit of the EPS VB113 by HPLC analysis. Figure 2 shows the HPLC chromatogram of EPS VB113 in comparison to the standard sugars. As can be seen, a very clear single peak assigned to the glucose in the standards was observed for the monomer profile of strain VB113, demonstrating that EPS VB113 was a homopolymeric α-glucan producer. Previous studies showed not only α-glucan production as a homopolymeric EPS [9] but also a levan-type homopolymeric one for *Leuconostoc mesenteroides* strains [17], while for strain VB113, only α-glucan production was observed under the tested culture conditions.

**Figure 3:**
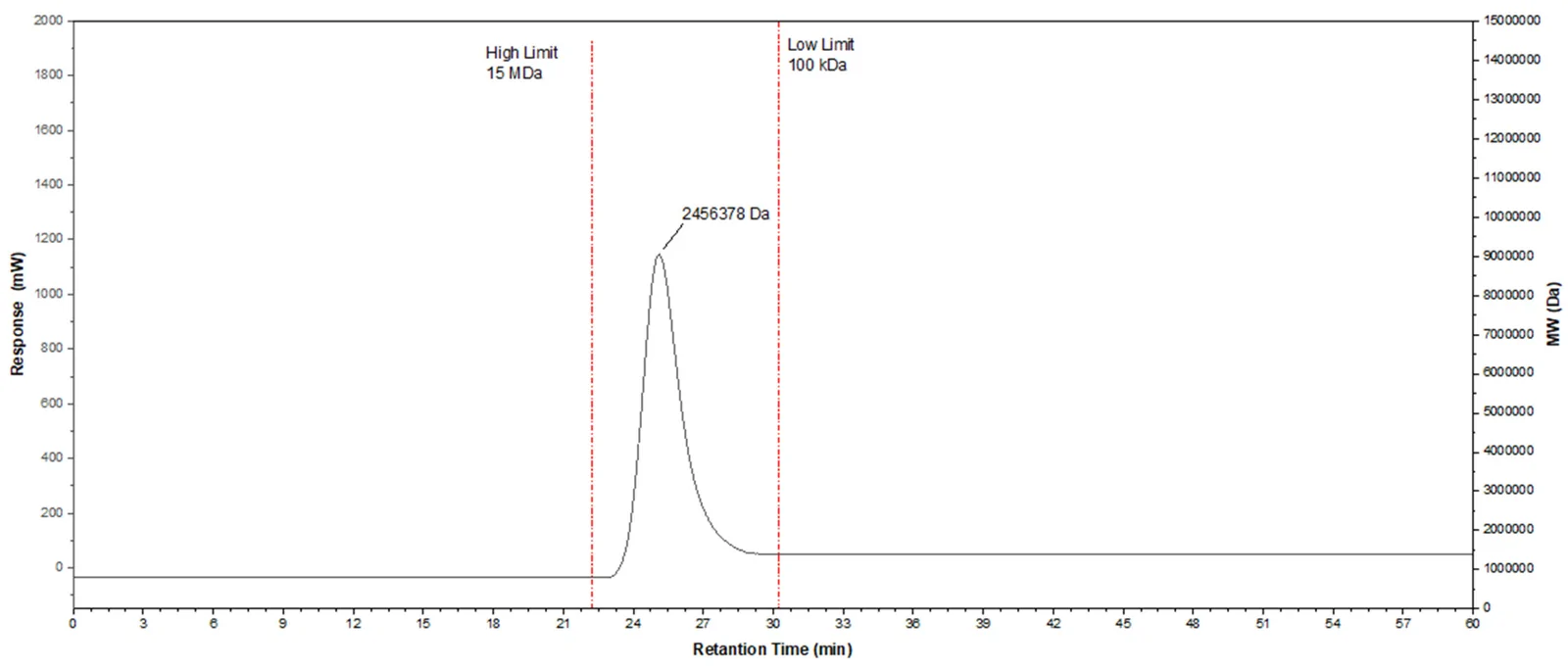
GPC analysis of glucan VB113 demonstrating it molecular weight.

Another important physicochemical characteristic of EPSs is their molecular weight, and from this perspective, the molecular weight of glucan VB113 was determined by GPC analysis. As can be seen in Figure 3, glucan VB113 demonstrated a molecular weight of ~ 2.5 × 10^6^ Da. This level of molecular weight was similar to the previous observations [1,18], although higher levels of molecular weights for other glucans were also reported [19,20]. Nevertheless, glucan VB113 demonstrated an average level of molecular weight in terms of LAB EPSs, including glucan, suggesting its potential for different physicochemical functionalities.

### 2.3. Chemical Characterization of Glucan VB113 by NMR Analysis

**Figure 4:**
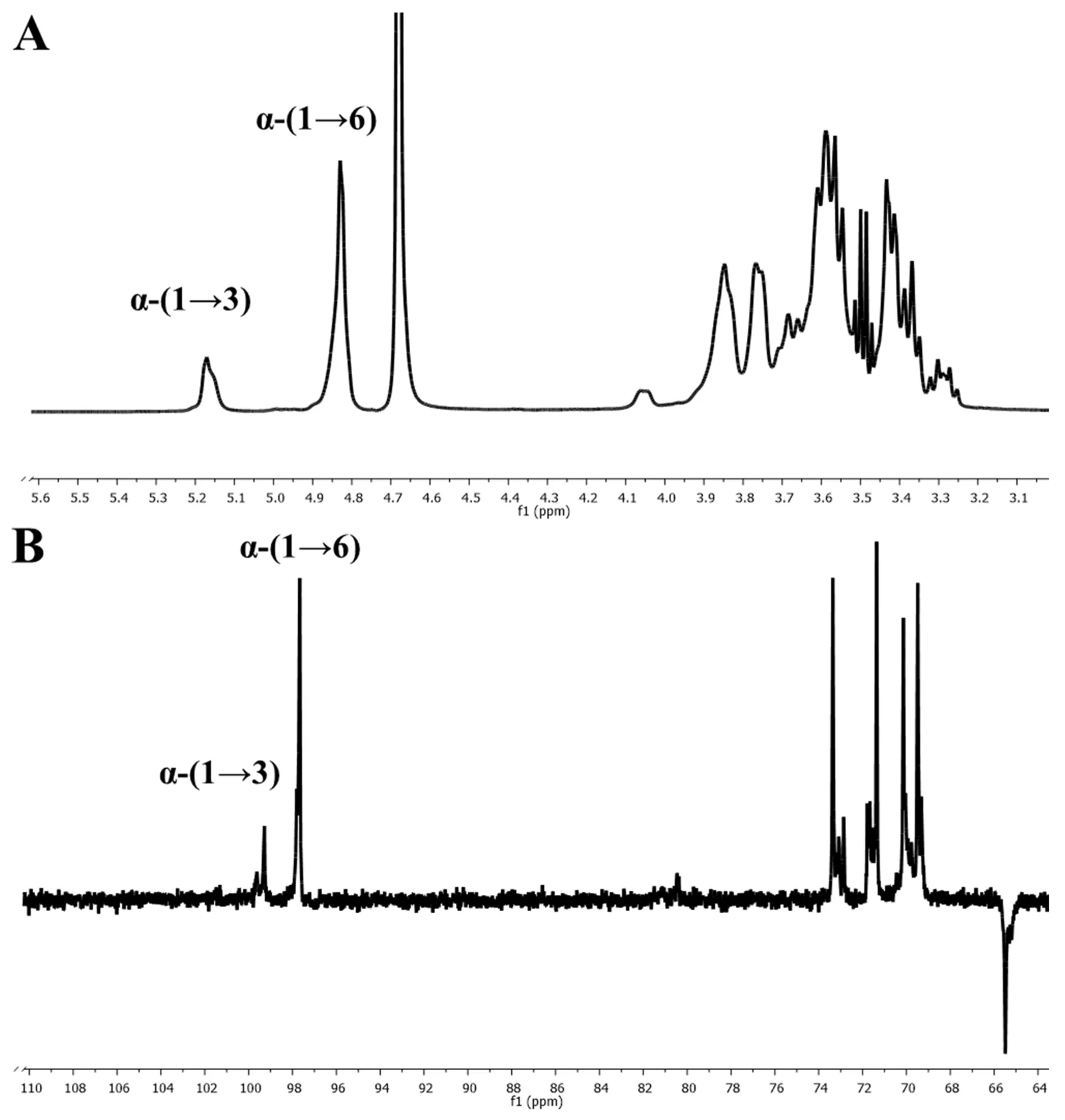
^1^H NMR (**A**) and ^13^C NMR (**B**) spectra of dextran VB113 produced by *Leuconostoc mesenteroides* VB113.

The structural formation of glucan VB113 was further determined by ^1^H and ^13^C NMR analysis, and Figure 4A,B demonstrate the ^1^H and ^13^C NMR data of glucan VB113, respectively. As can be seen in Figure 4B, typical spectral resonances at around 3.2 ppm to 5.2 ppm were observed, originating from the ring proton and the anomeric regions, as previously described [9]. For the anomeric region, two spectral resonances were observed at around 4.83 ppm and 5.18 ppm, which were previously assigned to (1 → 6)-linked α-D-glucose units and (1 → 3)-linked α-D-glucose units, respectively [1,9]. Importantly, the proportion of the (1 → 6)-linked α-D-glucose units to (1 → 3)-linked α-D-glucose units was 4:1, suggesting a highly branched structure for dextran VB113. The ^13^C NMR spectra of dextran VB113 were compatible with the ^1^H data demonstrating (1 → 3)-linked α-D-glucose units and (1 → 6)-linked α-D-glucose units at 99.75 ppm and 97.7 ppm for the anomeric C, respectively [9], and peaks C-2 and C-6 were observed in the regions of 73.5, 71.2, 69.9, 69.5 and 65.7 ppm [1,9].

These findings originating from ^1^H and ^13^C NMR data demonstrate that *Leuconostoc mesenteroides* VB113 was capable of the production of a branched dextran structure with 20% (1 → 3)-linked α-D-glucose units. Previous studies also demonstrated the dextran-type homopolymeric EPS production of *Leuconostoc mesenteroides* strains, and, in general, the level of (1 → 3)-linked α-D-glucose units was observed to be within ~5% [9,21], although similar to our observation, highly branched dextran was also characterized for this species [22]. The highly branched nature of dextran VB113 suggests that every 1000 glucose units within the structure formed 200 branch linkages, as discussed earlier [23], and this chemical nature might affect its final technological and functional roles [24]. These findings underscore the necessity of isolation of LAB from non-conventional ecological niches to discover novel wild-type strains. Such environments serve as valuable reservoirs for strains possessing unique metabolic profiles, as demonstrated by strain VB113, which synthesizes a structurally distinct, highly branched dextran.

### 2.4. FT-IR Analysis of Dextran VB113

**Figure 5:**
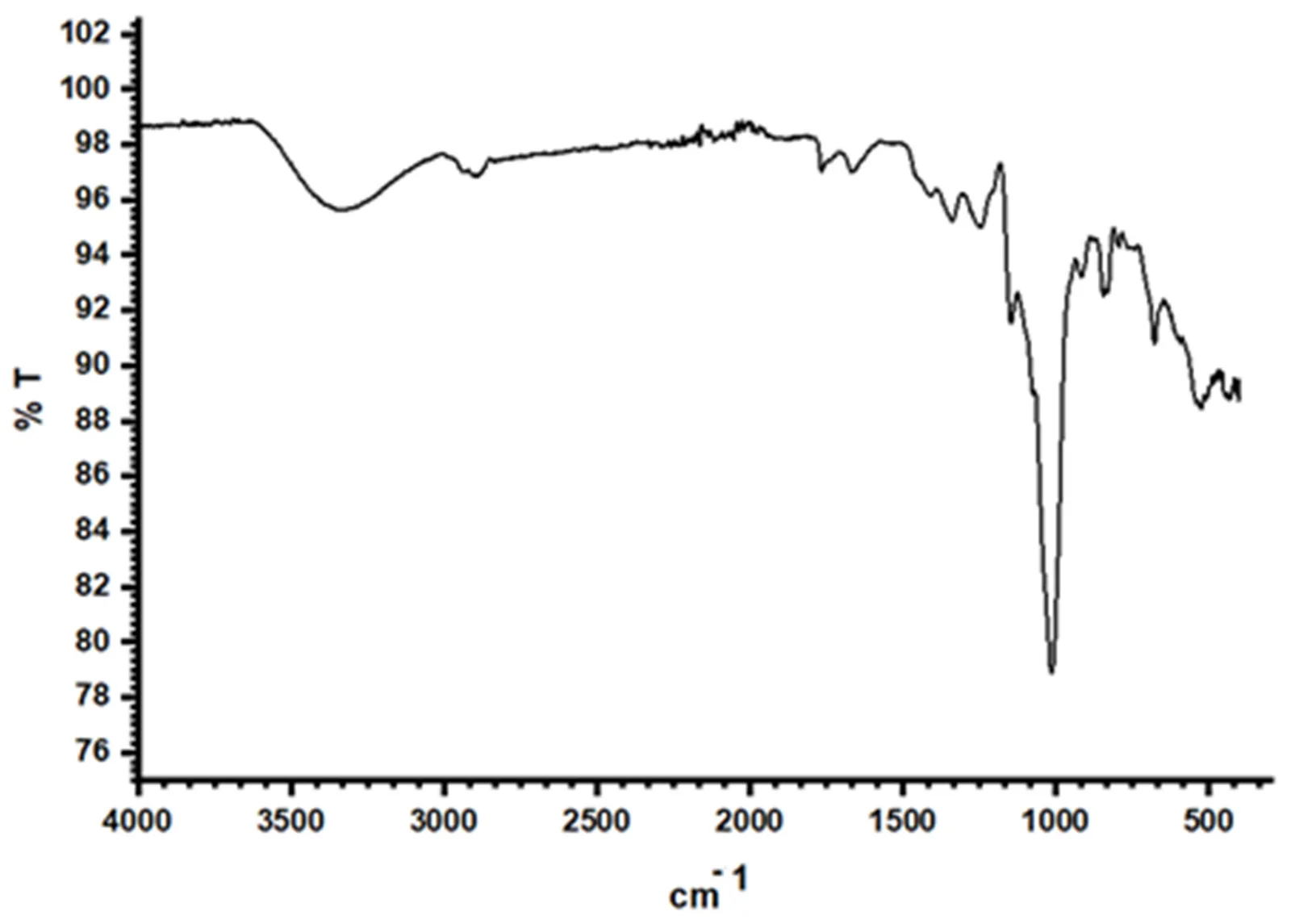
FTIR spectra of dextran VB113 demonstrating the functional groups in the structure obtained within 400–4000 cm^−1^ range.

In order to determine the presence of specific functional groups within the structure of dextran VB113, FTIR analysis was applied and the spectra were recorded in the range of 4000–400 cm^−1^ (Figure 5). Similar to the previous observations, a typical polysaccharide peak was obtained within the 2900–3500 cm^−1^ region, originating from the hydroxyl groups of dextran VB113 [1,25].

A relatively wide and small peak was observed at around 2925 cm^−1^ and 1645 cm^−1^, which were previously assigned to the C-H bond stretching and vibrations of carboxylic groups within the sugar ring, respectively [1,26]. In the fingerprint region, an intense peak between 950 cm^−1^ and 1200 cm^−1^ with a maximum absorbance observed at 1012 cm^−1^ revealed the stretching vibrations of the C-O-C glycosidic bond bridge alongside C-O stretching at the ring positions [1]. Importantly, a weak band in the anomeric region at approximately 845 cm^−1^ was observed, which was previously assigned to the α-linked D-glucopyranose units [13], suggesting that the tested polymer was a glucan-type homopolysaccharide. These findings, together with the NMR data, confirm the structure of EPS VB113 as a dextran-type homopolysaccharide harboring (1 → 3)-linked α-D-glucose units.

### 2.5. Thermal Characteristics of Dextran VB113

**Figure 6:**
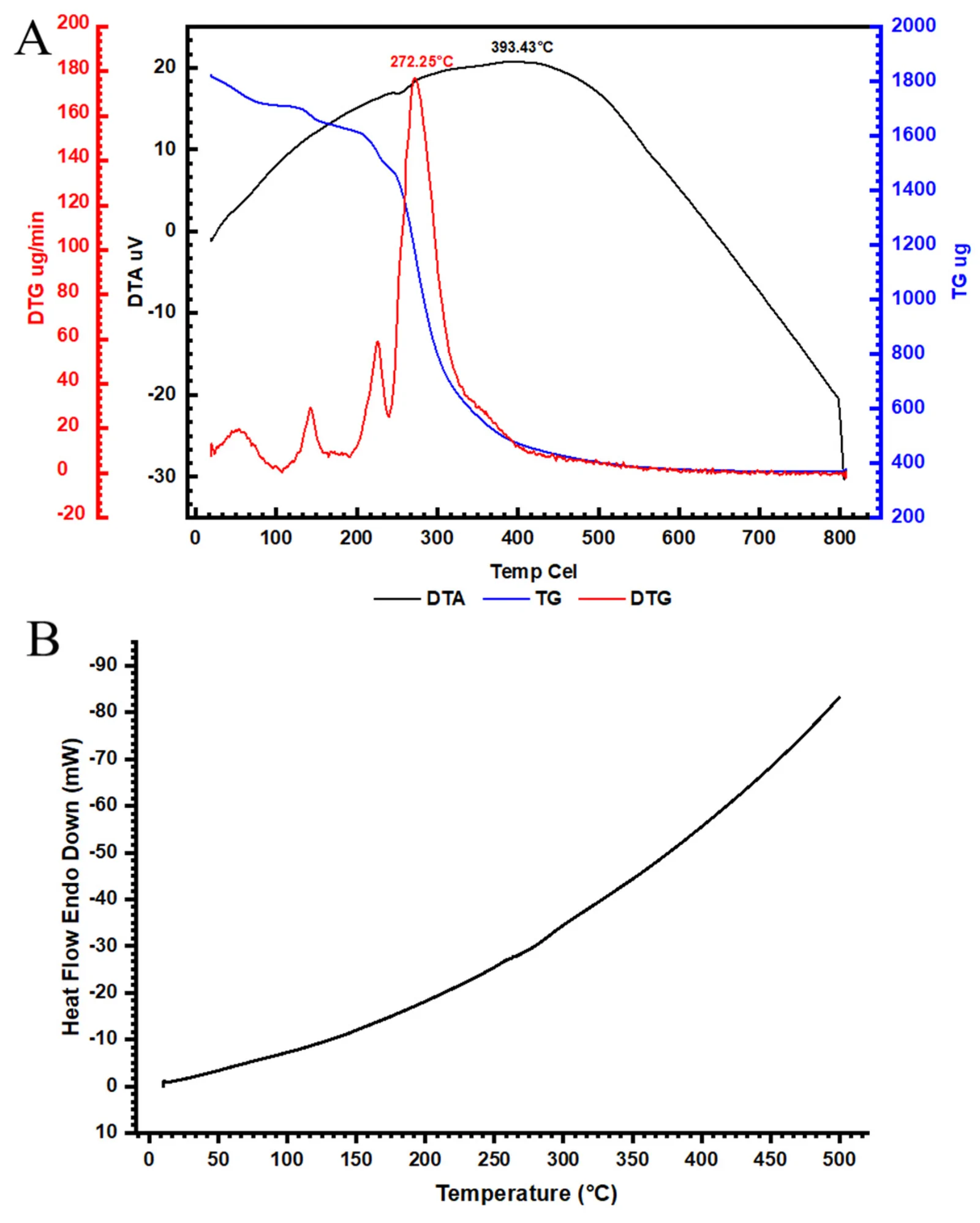
TG/DTG (**A**) and DSC (**B**) thermograms of dextran VB113 demonstrating its high thermal stability.

Industrial applications, especially for the food industry, require important levels of thermal applications, and biopolymers such as α-glucans from LAB demonstrate a certain level of thermal resistance, making these polymers unique for different technological and functional roles. In this respect, the thermal features of dextran VB113 were determined by TGA and DSC analyses (Figure 6). As can be seen in Figure 6A showing the TG/DTG profile of dextran VB113, a three-step degradation profile was observed, starting with moisture desorption up to 120 °C, followed by a mild degradation phase up to 240 °C and a final primary degradation phase observed between 240 and 350 °C with a prominent DTG peak at 272.25 °C. In the moisture desorption stage, a weight loss of 9.4% was observed, whereas in the primary degradation phase, 67.2% mass loss was observed. This sharp degradation phase was suggested to be related to the cleavage of glycosidic linkages, depolymerization, and the decarboxylation of carbon chains [27]. After 400 °C, a gradual mass loss of around 2.2% was observed, suggesting the slow carbonization and ash formation of the residual organic matter. For the DTA data, an initial endothermic trend up to 100 °C was observed, which was followed by a major exothermic transition peak broadly at 393.43 °C, suggesting thermo-oxidative degradation. The TGA data of dextran VB113 was similar to previous observations with low levels of branched glucans [1,25], suggesting its suitability for high-thermal applications. In a previous study, a highly branched dextran (~50%) revealed a degradation temperature of 289 °C [7], which is also compatible with the thermal stability of dextran VB113. Figure 6B demonstrates the DSC profile of dextran VB113, showing a smooth degradation process within 0–500 °C of heat application [1], and this profile is inconsistent with the TGA data. Together with the DSC and TGA data, dextran VB113 revealed a highly resistant thermal profile with 272.25 °C as the primary degradation temperature, which suggests its potential functionality for high-thermal applications in food and other related industries.

### 2.6. Physical Status of Dextran VB113 Determined by XRD Analysis

**Figure 7:**
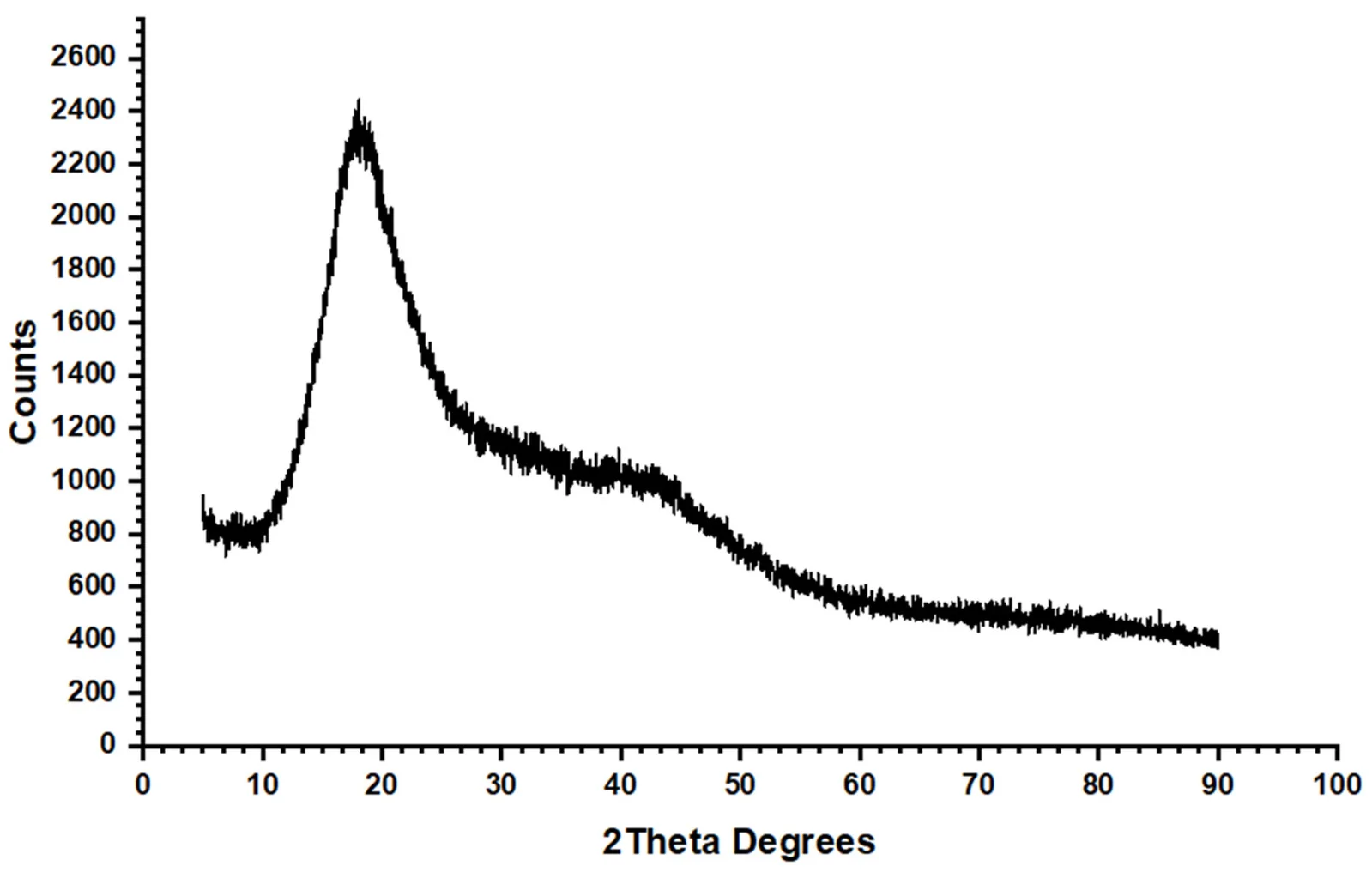
XRD chromatogram of glucan VB113 showing its amorphous nature.

The physical status of biopolymers might be important for their final technological and functional roles [25], and in this respect, XRD analysis was applied to unveil the crystallographic characteristics of dextran VB113. As can be seen in Figure 7, dextran VB113 demonstrated a completely amorphous morphology with characteristics of disordered macromolecular arrangements [20,25]. Previously, the amorphous forms were suggested to demonstrate enhanced solubility that would potentially affect the biological activity of dextran VB113 [25,28]. It should also be noted that α-glucans from LAB strains can demonstrate a crystalline [19], semi-crystalline [1] or amorphous [20] nature, suggesting the strain-specific role of the final physical status of the produced EPS. Overall, XRD data revealed the amorphous status of glucan VB113, which can be related mainly to its strong solubility potential and enhanced biological activity such as antioxidant capacity.

### 2.7. Surface Topography of Dextran VB113 Tested by Atomic Force Microscopy Analysis

**Figure 8:**
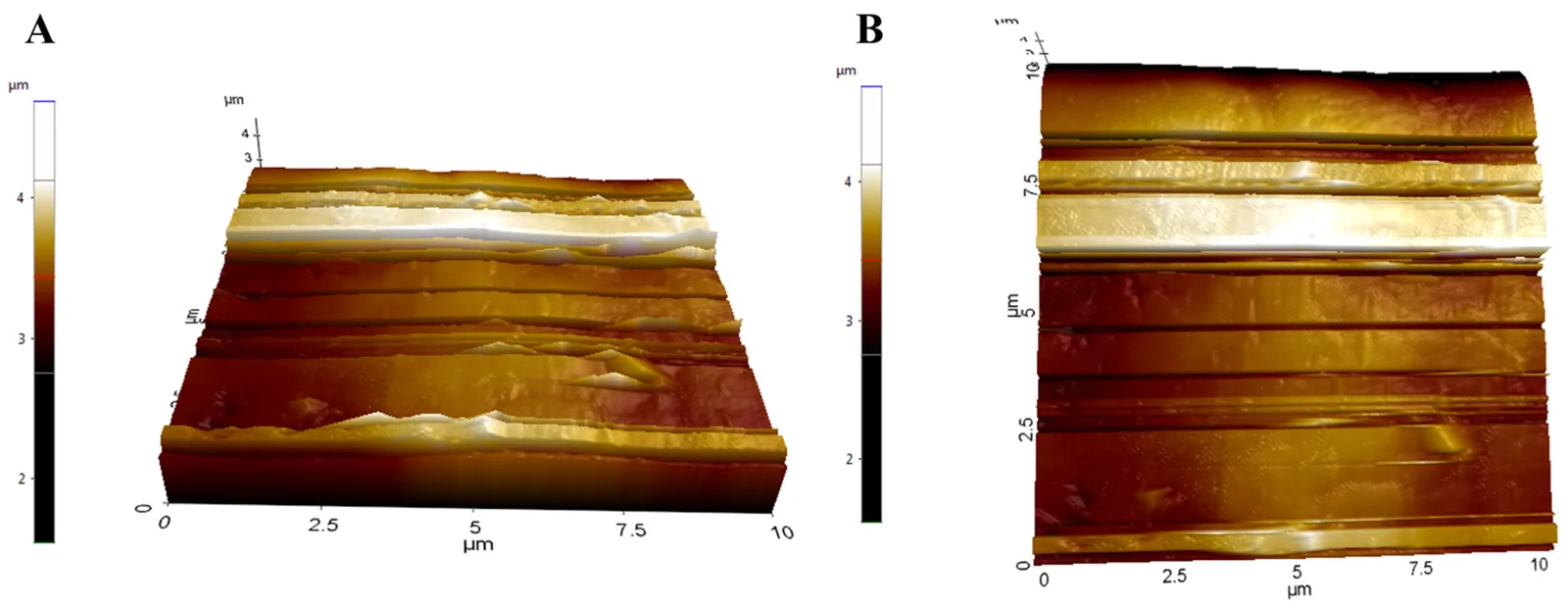
Three-dimensional topography (**A**) and 2D top-down height projection (**B**) of the aqueous solution of glucan VB113.

The surface nanostructure and topographical morphology of dextran VB113 were determined by AFM analysis, and Figure 8A,B show the 3D topography and 2D top-down height projection of the aqueous solution of dextran VB113 that was scanned after the air-drying of the solution at RT. The dextran VB113 revealed a fibrillary topology structure, and the distinct directional alignment potentially suggested an intermolecular hydrogen bonding interaction in the glucan matrix. This topographical matrix forming features of glucan VB113 might be important for its water holding characteristics, and having 20% (1 → 3)-linked α-D-glucose units as branches might help for the formation of this self-assembled matrix. Previous studies demonstrated distinct topographical morphologies for different glucans such as ellipsoidal, spheroidal or tangled networks like structural features [1,27], which suggest the importance of the final structural features in determining the surface morphology of the glucans.

### 2.8. Morphological Characteristics of Dextran VB113 Detected by Scanning Electron Microscopy

**Figure 9:**
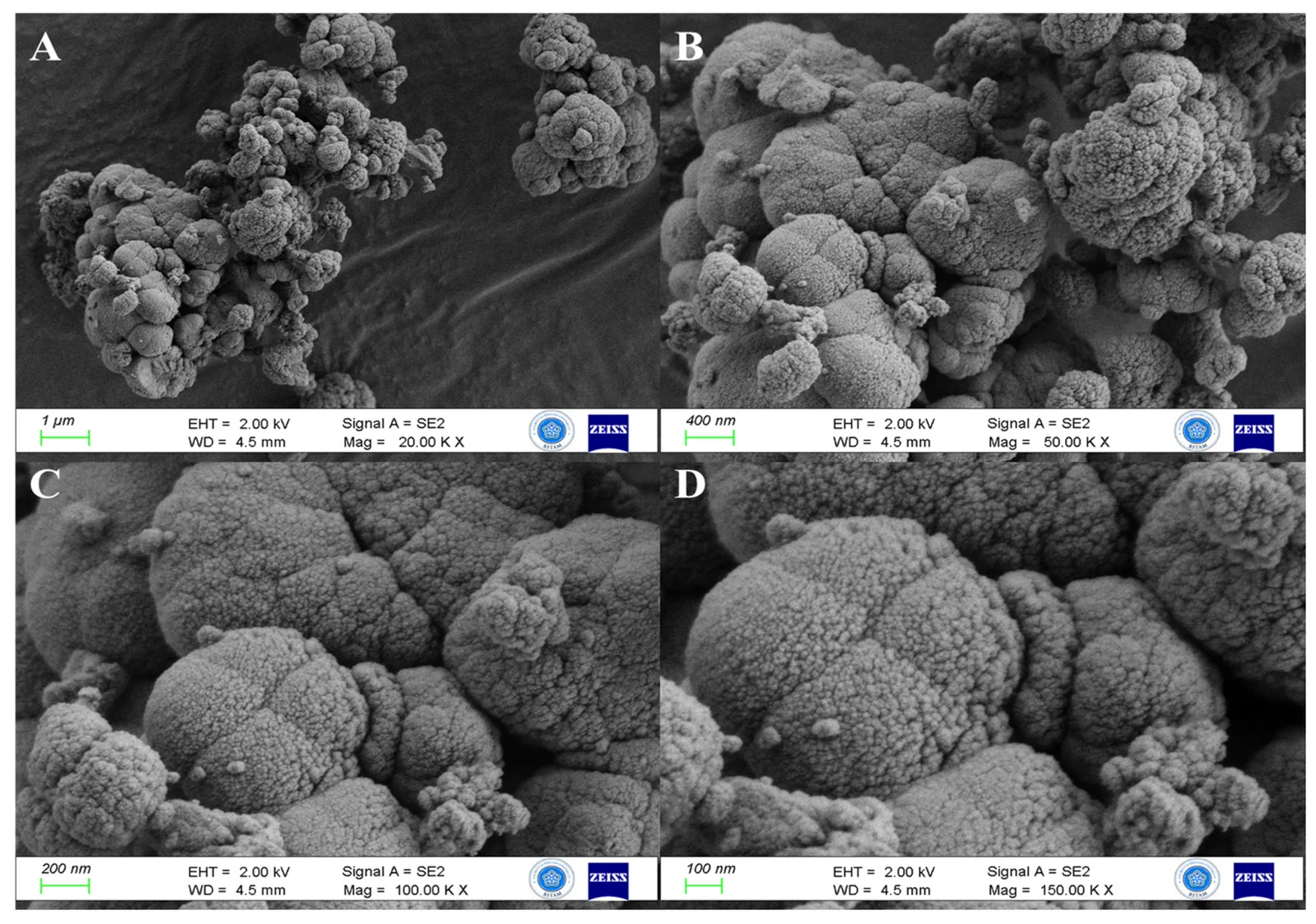
SEM images of the dextran VB113 at different magnification levels, 20,000× (**A**), 50,000× (**B**), 100,000× (**C**) and 150,000× (**D**), showing its surface micromorphology formed of uniform ultra-fine nanogranules.

The micro- and nanostructural characteristics of the dextran VB113 were evaluated by SEM analysis at various magnifications (Figure 9A–D). As can be seen at different magnifications, dextran VB113 demonstrated a characteristic hierarchical, cauliflower-like morphology composed of tightly clustered micro-spherical aggregates, and under high magnifications (Figure 9D), it can be observed that the surface of these micro-spherical aggregates was formed with uniform ultra-fine nanogranules. These nanostructural characteristics of the dextran VB113 might be important for network interactions, potentially making this polymer strongly available for distinct nanotechnology applications. Previous studies demonstrated distinct microstructural features for different glucans [1,19,22,25], and having a surface micromorphology formed of uniform ultra-fine nanogranules might be important for the technological and functional roles of glucan VB113.

## 3. Materials and Methods

### 3.1. Bacterial Strain and Testing Its EPS Production Under Sucrose Environment

In this study, *Leuconostoc mesenteroides* VB113, a previously identified potential EPS-producing LAB strain isolated from traditional Caucasian Pear (*Pyrus communis* subsp. *caucasica*) pickle samples, was evaluated for its EPS production characteristics [1]. This strain was propagated in De Man, Rogosa and Sharpe (MRS) medium at 30 °C for 2 days under anaerobic conditions for standard growth. Following the two constitutive growth periods in 20 mL MRS medium, the strain was then streaked into BHI agar with or without 10% sucrose to visualize its EPS production properties. The plates were incubated at 30 °C for 2 days and strain morphology was observed.

### 3.2. Determination of the Repeating Unit Structure and Molecular Weight of EPS VB113

Following the confirmation of the EPS production characteristics of *Leuconostoc mesenteroides* VB113, this strain was further grown in modified BHI medium (no yeast extract and containing 2% sucrose, calf brains 12.5 g/L, beef heart 5 g/L, glucose 10 g/L, disodium hydrogen phosphate 2.5 g/L, peptone 10 g/L, sodium chloride 5 g/L, Sucrose 20 g/L, Tween 20 1 g/L, Sodium acetate 5 g/L, Peptone casein 5 g/L, Peptone meat 5 g/L and Magnesium Sulfate 0.2 g/L) at 30 °C for 24 h to extract its EPS from the culture supernatant [29]. Details of the extraction and partial purification process using a 12,000–14,000 Da membrane were given previously [29], and the final purified EPS was obtained by lyophilization and subjected to further characterization tests. For the dialysis, EPS was held in the 12,000–14,000 Da membrane against UP water at 4 °C for 72 h with 2 dialysate replacements per 24 h.

At first, the repeating unit structure of EPS VB113 was determined by HPLC analysis [1], and for this, EPS VB113 was hydrolyzed using perchloric acid (70%) (80 °C, 1 h) and the solution was neutralized using 5 M KOH. A Shimadzu HPLC system with an RID-10A detector was used for sugar monomer detection under the conditions of CARBOsep CHO-682 Pb Column, 25 °C column temperature, and H_2_O with a flow rate of 0.7 mL min^−1^ as the mobile phase. Standard sugars of glucose, maltose, fructose and galactose were used during analysis. The HPLC system was also used for the determination of the molecular weight of EPS VB113, and for this, a PolySep-GFC-P 6000 (LC Column 300 × 7.8 mm) (Phenomenex) column was used.

### 3.3. Characterization of Core Structure of EPS VB113 by NMR Analysis

The structural characterization of the EPS VB113 was carried out by ^1^H and ^13^C NMR spectroscopy analysis, and for this, an Agilent 400 MHz NMR instrument was used. Details of the NMR analysis are given elsewhere [29].

### 3.4. Characterization of Functional Groups of EPS VB113 by FTIR Spectroscopy Analysis

The functional groups within the EPS VB113 were determined by an FTIR spectrophotometer (Perkin Elmer Spectrum 65), and a region of 4000–400 cm^−1^ was scanned at a spectral resolution of 4 cm^−1^ in transmission mode. FTIR spectra were recorded [1].

### 3.5. Characterization of Thermal Properties of EPS VB113 by TGA and DSC Analysis

For the determination of the thermal characteristics of EPS VB133, TGA and DSC analyses were applied. For TGA analysis, a thermal analyzer (EXSTAR 7300, SII Nanotechnology) was used to determine the thermogravimetric loss, and EPS VB113 was subjected to a heating process of 25–800 °C temperature under a nitrogen atmosphere with a heating ramp of 10 °C min^−1^ [1]. For DSC analysis, a previously described methodology was used [29].

### 3.6. Characterization of Crystallographic Properties of EPS VB113 Detected by XRD Analysis

XRD analysis was applied to test the physical status of the EPS VB113, and for this, a Bruker D8 Discover instrument was used [29]. To obtain the XRD diffractogram, Cu Kα radiation and a Ni filter with generator settings of 40 kV, 40 mA, were utilized with Breg Brentano θ:2θ geometry in the range of 5–90° and a 0.03° increment in step size.

### 3.7. Characterization of Morphological Properties of EPS VB113 Detected by SEM and AFM Analysis

For the determination of the surface characteristics of EPS VB113, AFM analysis was applied using an AFM PLUS+ (NanoMagnetic) instrument. The AFM analysis of EPS VB113 was performed using previously described methodology [17]. For the microstructural characterization of EPS VB113, SEM analysis was applied with a Zeiss instrument at an accelerating voltage of 2.0 kV, and images were taken at different magnification levels.

## 4. Conclusions

Metabolites of LAB have gained special interest following the introduction of postbiotics terminology, and homopolymeric EPSs are key postbiotic metabolites of LAB that can be produced in distinct structural characteristics. In this study, *Leuconostoc mesenteroides* VB113 was isolated from a traditional pickle environment as a homopolymeric EPS producer and its physicochemical characteristics were determined. Structural characterization demonstrated that strain VB113 produced a highly branched dextran with 20% levels of (1 → 3)-linked α-D-glucose units, and the molecular weight of glucan VB113 was 2.4 × 10^6^ Da. A high level of thermal stability was observed for dextran VB113, with a primary degradation phase noted at 272.25 °C. This amorphous glucan was further tested for its microstructural features and demonstrated characteristic hierarchical, cauliflower-like morphology composed of tightly clustered micro-spherical aggregates formed of uniform ultra-fine nanogranules. These characteristics of dextran VB113 suggest its potential for future applications for technological or functional purposes such as utilization as a biothickener agent or for its biological activity. The branched characteristics of VB113, together with its high molecular weight, suggest its function as a potential fat replacer in low-calorie dairy/plant-based meat analogs and gelling agents in fermented plant matrices. The high thermal stability and nanostructure properties of VB113 also suggest its functionality for extruded formulations, as well as for encapsulation processes. The current study mainly focused on the structural and physicochemical characterization of dextran VB113, although more studies are required to test this glucan in real food formulations, as well as for in vitro and in vivo functionality studies such as antioxidant and prebiotic capacity.

## Data Availability

All the data are available in the article.
